# BMC *Neurology* reviewer acknowledgement 2014

**DOI:** 10.1186/s12883-015-0264-x

**Published:** 2015-02-13

**Authors:** Tim Shipley

**Affiliations:** BioMed Central, Floor 6, 236 Gray’s Inn Road, London, WC1X 8HB UK

## Abstract

The editors of BMC Neurology would like to thank all our reviewers who have contributed to the journal in Volume 14 (2014).

**Nicola Acciarri**

Italy

**Hiroaki Adachi**

Japan

**Harold Adams**

United States of America

**Anahita Adeli**

United States of America

**Federica Agosta**

Italy

**Aftab Ahmad**

Singapore

**Samrah Ahmed**

United Kingdom

**Jessica Ailani**

United States of America

**Gianluca Aimaretti**

Italy

**Faisal Al Otaibi**

Saudi Arabia

**Philipp Albrecht**

Germany

**Suvarna Alladi**

India

**Carl Allen**

United States of America

**Francisco Alvarez**

Spain

**Maria Amato**

Italy

**Norberto Andaluz**

United States of America

**Ilinca Andreea**

Sweden

**Marilou Andres**

United States of America

**Pasquale Annunziata**

Italy

**Antonio Arauz**

Mexico

**Agnieszka Ardelt**

United States of America

**Didem Arslantas**

Turkey

**Ayce Atalay**

Turkey

**Shahram Attarian**

France

**César Ávila**

Spain

**Richard Aviv**

Canada

**Eric Azabou**

France

**Umesh Badrising**

Netherlands

**Francesca Bagnato**

United States of America

**Jong Sam Baik**

Korea, South

**Konstantin Balashov**

United States of America

**Carolina Ballert**

Switzerland

**James Baraniuk**

United States of America

**Piero Barbanti**

Italy

**Iacopo Barbetta**

Italy

**Mark Baron**

United States of America

**Mera Barr**

Canada

**Henryk Barthel**

Germany

**Petra Bartova**

Czech Republic

**Amit Batla**

United Kingdom

**Amit Batra**

India

**Sachin Batra**

United States of America

**Kyra Becker**

United States of America

**Heleen Beckerman**

Netherlands

**Christian Beetz**

Germany

**Maria Isabel Behrens**

Chile

**Maria Donata Benedetti**

Italy

**Ralph Benedict**

United States of America

**Gary Bennett**

Canada

**Meriem Bensalem-Owen**

United States of America

**Alberto Benussi**

Italy

**Roberto Bergamaschi**

Italy

**Vance Berger**

United States of America

**Julie Bernhardt**

Australia

**Marcelo Berthier**

Spain

**Antonio Bertolotto**

Italy

**Roongroj Bhidayasiri**

Thailand

**Philippe Bijlenga**

Switzerland

**Andreas Binder**

Germany

**Patrícia Biselli-Chicote**

Brazil

**Sandra Black**

United States of America

**Ignacio Blanco**

Spain

**Andrew Blann**

United Kingdom

**Richard Bohannon**

United States of America

**Jacopo Bonavita**

Italy

**Mickael Bonnan**

France

**Michael Borich**

Canada

**Sevasti Bostantjopoulou**

Greece

**Aaron Boster**

United States of America

**Alex Bottle**

United Kingdom

**Marek Brabec**

Czech Republic

**Ari Breiner**

Canada

**Harald Breivik**

Norway

**Thomas Brinker**

United States of America

**Bruno Brochet**

France

**Amalia Cecilia Bruni**

Italy

**Melissa Buelow**

United States of America

**Paulo Bugalho**

Portugal

**Cheryl Bushnell**

United States of America

**Philippe Cabre**

Martinique

**Dominique Anne-Michele Cadilhac**

Australia

**Vittorio Calabrese**

Italy

**Alfred Callahan**

United States of America

**Jean-Philippe Camdessanche**

France

**Isabella Canavero**

Italy

**Patrícia Canhão**

Portugal

**Colleen Canning**

Australia

**Louis Caplan**

United States of America

**Marco Capobianco**

Italy

**Roberto Cappellani**

United States of America

**Roberto Cappellani**

Italy

**Andrew Carlson**

United States of America

**Mauro Giovanni Carta**

Italy

**Carlo Casali**

Italy

**Ilaria Casetta**

Italy

**Marco Castori**

Italy

**James Cauraugh**

United States of America

**Juan Cebral**

United States of America

**Elisabeth Gulowsen Celius**

Norway

**David Cella**

United States of America

**Diego Centonze**

Italy

**Baidarbhi Chakraborty**

India

**Kuo-Hsuan Chang**

Taiwan

**Márcia Lorena Chaves**

Brazil

**Bingkun Chen**

United States of America

**Jack Chen**

United States of America

**Chih-Hung Chen**

Taiwan

**Hsiu-Ling Chen**

Taiwan

**Yu-Wei Chen**

Taiwan

**Shankar Chinta**

United States of America

**Yong Won Cho**

Korea, South

**Muhammad Chohan**

United States of America

**Christian Fynbo Christiansen**

Denmark

**Jordi Clarimon**

Spain

**Daniel Clayton**

United States of America

**Geoff Cohen**

United Kingdom

**William Copen**

United States of America

**Gianluca Coppola**

Italy

**Ester Coutinho**

Portugal

**Jonathan Coutinho**

Netherlands

**Carlos Cruchaga**

United States of America

**Nicolas Cuenca**

Spain

**Toby Cumming**

Australia

**Elisa D'Agati**

Italy

**Bernard Dan**

Belgium

**Shprecher David**

United States of America

**Joanne Davidson**

New Zealand

**Hayk Davtyan**

United States of America

**Michael Dayan**

United States of America

**Pasquale De Bonis**

Italy

**Veerle De Herdt**

Belgium

**Roberto De Masi**

Italy

**Aaron De Souza**

India

**Marianne De Visser**

Netherlands

**Elizabeth Dean**

Canada

**Andrea Debarber**

United States of America

**Gilles Defer**

France

**Pilar Delgado**

Spain

**Alessandro Della Puppa**

Italy

**Emilien Delmont**

France

**Arnaud Delval**

France

**Reinhard Dengler**

Germany

**Chantal Depondt**

Belgium

**Troy Desai**

United States of America

**David Devos**

France

**Haibo Di**

China

**Marco Dibonaventura**

United States of America

**Jennifer Diedler**

Germany

**Hans-Christoph Diener**

Germany

**Hu Ding**

China

**Gulden Diniz**

Turkey

**Diederik Dippel**

Netherlands

**Giulio Disanto**

United Kingdom

**Ondrej Dolezal**

United Kingdom

**Peter Donofrio**

United States of America

**Paul Dorian**

Canada

**Jan Dörr**

Germany

**Simone Dorsch**

Australia

**Jane Driver**

United States of America

**Andrew Ducruet**

United States of America

**Hugues Duffau**

France

**Kamil Duris**

Czech Republic

**Emmanuelle Duron**

France

**Siobhan Durran**

United Kingdom

**Aparna Dutt**

India

**Gammon Earhart**

United States of America

**Jodi Edwards**

Canada

**Filip Eftimov**

Netherlands

**Ulla Ellfolk**

Finland

**Catherine Elliott**

Australia

**Liene Elsone**

United Kingdom

**Gregory Enns**

United States of America

**Ricardo Erazo**

Chile

**Monica Falautano**

Italy

**Henrik Falhammar**

Sweden

**Cristian Falup-Pecurariu**

Romania

**Alfonso Fasano**

Canada

**Katharina Faust**

Germany

**Howard Federoff**

United States of America

**Seyed-Mohammad Fereshtehnejad**

Sweden

**Oscar Fernández**

Spain

**Israel Fernandez Cadenas**

Spain

**Davinia Fernández-Espejo**

Canada

**Henrique Ferraz**

Brazil

**Peter Feys**

Belgium

**Terry Fife**

United States of America

**Fernando Figueira**

Brazil

**Christian Foerch**

Germany

**Maria Joao Forjaz**

Spain

**Renerio Fraguas**

Brazil

**Marcondes França Jr**

Brazil

**Angela Frank-Briggs**

Nigeria

**Carolyn Fredericks**

United States of America

**Siqing Fu**

China

**Michitaka Funayama**

Japan

**Michael Gaab**

Germany

**Tereza Gabelic**

United States of America

**Salvatore Galati**

Switzerland

**Daniela Galimberti**

Italy

**Seana Gall**

Australia

**Eric Gamazon**

United States of America

**Diana Gavrila**

Spain

**Stefano Gazzina**

Italy

**David Geldmacher**

United States of America

**Henrik Gensicke**

Switzerland

**Florian Gessler**

Germany

**Othman Ghribi**

United States of America

**Olivier Godefroy**

France

**William Goggins**

Hong Kong

**Joana Gomes**

Portugal

**Francisco Gondim**

Brazil

**Benedetta Goretti**

Italy

**Harry Gould**

United States of America

**James Graham**

United States of America

**Dido Green**

United Kingdom

**Martin Griebe**

Germany

**Rohan Grimley**

Australia

**Bradley Gross**

United States of America

**Aihua Gu**

China

**Eric Guedj**

France

**Sasha Gulati**

Norway

**Yelena Guller**

United States of America

**Jose Gutierrez**

United States of America

**Mario Habek**

Croatia

**Monika Hackl**

Austria

**Shaheryar Hafeez**

United States of America

**Jesper Hagemeier**

United States of America

**Toby Hall**

Australia

**Thilo Hammen**

Germany

**Adam Handel**

United Kingdom

**Lahiru Handunnetthi**

United Kingdom

**Bjørge Herman Hansen**

Norway

**Jakob Møller Hansen**

Denmark

**Shuanglin Hao**

United States of America

**Ljubica Harhaji**

Serbia

**Jenni Harrison**

United Kingdom

**Stephanie Harrison**

United Kingdom

**Richard Hartman**

United States of America

**Hans-Peter Hartung**

Germany

**Kazuko Hasegawa**

Japan

**Mazyar Hashemilar**

Iran

**Ritsuo Hashimoto**

Japan

**Shintaro Hayashi**

Japan

**Hrvoje Hecimovic**

Croatia

**Peter Hedera**

United States of America

**Karl Herrup**

United States of America

**Håkon Hofstad**

Norway

**Kazuhiro Hongo**

Japan

**Dana Horakova**

Czech Republic

**Michael Hornberger**

Australia

**Thomas Hornby**

United States of America

**Dismand Houinato**

Benin

**Henry Houlden**

United Kingdom

**Cheng-Yang Hsieh**

Taiwan

**Sharpley Hsieh**

Australia

**Gavin Hudson**

United Kingdom

**Branko Huisa**

United States of America

**Árpád Illés**

Hungary

**Masatsune Ishikawa**

Japan

**Hiroyuki Ishiura**

Japan

**Michael James**

United States of America

**Douglas Jeffery**

United States of America

**Kurt Jellinger**

Austria

**Jiann-Shing Jeng**

Taiwan

**Beom S. Jeon**

Korea, South

**Seul-Ki Jeong**

Korea, South

**Robert Jerskey**

United States of America

**Agulla Freire Jesus**

Spain

**Jianping Jia**

China

**Yuhua Jiang**

China

**Glen Jickling**

United States of America

**Byung Kwan Jin**

Korea, South

**Yan Jin**

United States of America

**Dong-Gyu Jo**

Korea, South

**James F Jones**

United States of America

**Stephanie Jones**

United Kingdom

**Gwo-Ping Jong**

Taiwan

**Peter Jongen**

Netherlands

**Kai Kaila**

Finland

**Marios Kaliakatsos**

United Kingdom

**Chandrasekaran Kaliaperumal**

United Kingdom

**Tomas Kalincik**

Australia

**Christian Kamm**

Switzerland

**Takashi Kanda**

Japan

**Kyusik Kang**

Korea, South

**Peter Kang**

United States of America

**Hiroshi Kanno**

Japan

**Zuleyha Karaca**

Turkey

**Lakshmi Sudha Karanam**

India

**Niki Karavitaki**

United Kingdom

**Jan Kassubek**

Germany

**Mahesh Kate**

Canada

**Zaza Katsarava**

Germany

**Michal Katz-Leurer**

Israel

**Katelyn Kavak**

United States of America

**Pawel Kawalec**

Poland

**Hugh Kearney**

United Kingdom

**Richard Keep**

United States of America

**Patrick Kehoe**

United Kingdom

**Christoph Kellinghaus**

Germany

**Andrew Kelso**

United Kingdom

**Jeff Kennedy**

United States of America

**Jürg Kesselring**

Switzerland

**Ali Khan**

Canada

**Dheeraj Khurana**

India

**Alice Kiger**

United Kingdom

**Ken-Ichiro Kikuta**

Japan

**Sung-Woon Kim**

Korea, South

**Dong-Eog Kim**

Korea, South

**Kwang-Sik Kim**

United States of America

**Seung-Ki Kim**

Korea, South

**Sang Jeong Kim**

Korea, South

**Vasilios Kimiskidis**

Greece

**Victor Kiri United**

Kingdom

**Fenella Kirkham**

United Kingdom

**Brad Klein**

United States of America

**Kleopas Kleopa**

Cyprus

**Anne Kloos**

United States of America

**Ian Kneebone**

Australia

**Sebastian Koch**

United States of America

**Matthew Koenig**

United States of America

**Vladimir Kostic**

Serbia

**Tibor Kovács**

Hungary

**Paul Krafft**

United States of America

**Peter Kraft**

Germany

**Michal Král**

Czech Republic

**Christine Kremer**

Sweden

**Rita Krishnamurthi**

New Zealand

**Hiraku Kumamaru**

United States of America

**Kishore Raj Kumar**

Australia

**Akira Kunimatsu**

Japan

**Yu-Min Kuo**

Taiwan

**Satoshi Kuroda**

Japan

**Suzanne Kuys**

Australia

**Andreas Kyrozis**

Greece

**Jan Laczó**

Czech Republic

**Kamakshi Lakshminarayan**

United States of America

**Yair Lampl**

Israel

**Anne-Marie Landtblom**

Sweden

**Silvia Lanfranconi**

taly

**Andrew Larner**

United Kingdom

**Norman Latov**

United States of America

**Maggie Lawrence**

United Kingdom

**Emilie Le Rhun**

France

**Kyeong-Seok Lee**

Korea, South

**Tsong-Hai Lee**

Taiwan

**Ville Leinonen**

Finland

**Lorenzo Leoncini**

Italy

**Andrew Levine**

United States of America

**Simon Lewis**

Australia

**Frank Leypoldt**

Germany

**Han Liang**

China

**Christina Lill**

Germany

**Shen-Yang Lim**

Malaysia

**Thien Thien Lim**

Malaysia

**Huey-Juan Lin**

Taiwan

**Keh-Chung Lin**

Taiwan

**Richard Lindley**

Australia

**Joan Lindsay**

Canada

**Sally Lindsay**

Canada

**Vasileios-Arsenios Lioutas**

United States of America

**Xu-Sheng Liu**

China

**Piergiorgio Lochner**

Italy

**Praween Lolekha**

Thailand

**Svetlana Lorenzano**

United States of America

**Cheng-Hsien Lu**

Taiwan

**Rimas Lukas**

United States of America

**Dorothée Lulé**

Germany

**Marion Luyat**

France

**Elizabeth Lynch**

Australia

**R Loch Macdonald**

Canada

**Antonella Macerollo**

United Kingdom

**Amir Hadi Maghzi**

Iran

**Klaus H. Maier-Hein**

Germany

**Johannes Mair**

Austria

**Margaret Mak**

Hong Kong

**Keishi Makino**

Japan

**Désirée Maltais**

Canada

**Anatol Manaenko**

United States of America

**Victor Mark**

United States of America

**Aikaterini Markopoulou**

United States of America

**Ruth Marshall**

Australia

**Ramon Marti**

Spain

**Paula Martin**

United States of America

**Kenia Martínez**

Spain

**Sheryl Martin-Schild**

United States of America

**Miquel Àngel Mas**

Spain

**Ana Maseda**

Spain

**Suleiman Massarweh**

United States of America

**Shinji Matsuda**

Japan

**Muneaki Matsuo**

Japan

**Ryu Matsuo**

Japan

**Andre Maues De Paula**

France

**Anna Mayhew**

United Kingdom

**Stefano Mazzoleni**

Italy

**Jenny Mccleery**

United Kingdom

**Joanne Mclaurin**

Canada

**Amy Mcpherson**

Canada

**Stephen Meairs**

Germany

**Andreas Meisel**

Germany

**Bruno Meloni**

Australia

**Avner Meoded**

United States of America

**Atte Meretoja**

Australia

**David Meya**

Uganda

**Mike Meyer**

United States of America

**Styliani Milioti**

Greece

**Magali Millecamps**

Canada

**Zsofia Miltenyi**

Hungary

**Alireza Minagar**

United States of America

**Eneida Mioshi**

United Kingdom

**Eneida Mioshi**

Australia

**Shahnaz Miri**

Iran

**Sonoko Misawa**

Japan

**Masahiro Mishina**

Japan

**Ushakant Misra**

India

**Hiroshi Mitoma**

Japan

**Nicola Modugno**

Italy

**German Moris**

Spain

**Reg Morris**

United Kingdom

**Brian Moseley**

United States of America

**Ian Mosley**

Australia

**Joe Moxon**

Australia

**Julia Muenchhoff**

Australia

**Shunji Mugikura**

Japan

**Martijn Muller**

United States of America

**Antonio Muscari**

Italy

**Massimo Musicco**

Italy

**Vitaliano Francesco Muzii**

Italy

**Dattatraya Muzumdar**

India

**Halvor Naess**

Norway

**Yoshinari Nagakane**

Australia

**Stephanie Nahas**

United States of America

**Robert Naismith**

United States of America

**Laleh Najafizadeh**

United States of America

**Makoto Nakajima**

Japan

**Taizen Nakase**

Japan

**Kaavya Narasimhalu**

Singapore

**Hiroto Narimatsu**

Japan

**Wassim Nasreddine**

Lebanon

**Kiumarss Nasseri**

United States of America

**Merrilee Needham**

Australia

**Aidan Neligan**

United Kingdom

**Edin Nevzati**

Switzerland

**Peter New**

Australia

**Nam Nguyen**

United States of America

**Garth Nicholson**

Australia

**Ichizo Nishino**

Japan

**Eduardo Nobile-Orazio**

Italy

**Anna Noel-Storr**

United Kingdom

**Bardia Nourbakhsh**

United States of America

**Kazuhiko Nozaki**

Japan

**George Ntaios**

Greece

**Eric Nussbaum**

United States of America

**Paul Nyquist**

United States of America

**Petra Nytrova**

Czech Republic

**Rónán O'Caoimh**

Ireland

**Fumiharu Ohka**

Japan

**Yasuyuki Okuma**

Japan

**Francesco Oliva**

Italy

**Robert Olson**

Canada

**Hiroaki Ooboshi**

Japan

**Antonio Orlacchio**

Italy

**Erol Ozan**

Turkey

**Julia Pakpoor**

United Kingdom

**Prakash Paliwal**

Singapore

**Yuesong Pan**

China

**Stefano Paolucci**

Italy

**Theodora Pappa**

Greece

**Chang-Hyun Park**

Korea, South

**Elena Parrini**

Italy

**Elizabeth Pathak**

United States of America

**Susann Patschan**

Germany

**Francesco Patti**

Italy

**Steven Pavlakis**

United States of America

**Siddharama Pawate**

United States of America

**Costanza Pazzaglia**

Italy

**Leema Reddy Peddareddygari**

United States of America

**Istvan Pelczer**

United States of America

**Alex Perchuk**

United States of America

**Santiago Perez-Lloret**

France

**Joel Perlmutter**

United States of America

**Lin Perry**

Australia

**Hélène Pessah-Rasmussen**

Sweden

**Maurizio Petrarca**

Italy

**Pietro Pietrini**

Italy

**Andrea Pilotto**

Italy

**Elmar Hans Pinkhardt**

Germany

**Anna Poggesi**

Italy

**Michel Pohl**

France

**Patricia Pohl**

United States of America

**Francesco Pontieri**

Italy

**Greg Pontone**

United States of America

**Marcel Post**

Netherlands

**Luca Prosperini**

Italy

**Janet Prvu Bettger**

United States of America

**Suresh Pujar**

United Kingdom

**Michael Pulley**

United States of America

**Andreas Puschmann**

Sweden

**Jukka Putaala**

Finland

**Koen Putman**

Belgium

**Norman Putzki**

Switzerland

**Gail Pyne-Geithman**

United States of America

**Song Qu**

China

**Carlo Cosimo Quattrocchi**

Italy

**Adam Quick**

United States of America

**Terry Quinn**

United Kingdom

**David Raffelt**

Australia

**Paolo Ragonese**

Italy

**Ralph Rahme**

United States of America

**Goran Rakocevic**

United States of America

**Nandhagopal Ramachandiran**

Oman

**Sreeram Ramagopalan**

United Kingdom

**Deepa Ramasamy**

United States of America

**Stefan Rampp**

Germany

**Neal Rao**

United States of America

**Lawrence Recht**

United States of America

**Ana Recober**

United States of America

**Gauthier Remiche**

Belgium

**Florence Riant**

France

**Iolanda Riba**

Spain

**Stephane Richard-Devantoy**

Canada

**Michael Riley**

United States of America

**Richard Rison**

United States of America

**Marco Riva**

Italy

**Jessica Robinson-Papp**

United States of America

**Maria Rocca**

Italy

**Annie Rochette**

Canada

**Constantin Roder**

Germany

**Helen Rodgers**

United Kingdom

**Chaturaka Rodrigo**

Sri Lanka

**David Rodriguez-Luna**

Spain

**Mayela Rodríguez-Violante**

Mexico

**Asuncion Romero - Molina**

Spain

**David Rosenthal**

United States of America

**Homayoun Roshanisefat**

Sweden

**Thomas Rossor**

United Kingdom

**Marco Rovaris**

Italy

**Lewis P. Rowland**

United States of America

**Sean Ruland**

United States of America

**Devon Ryan**

Germany

**Juan Saavedra**

United States of America

**Behnam Sabayan**

Netherlands

**Mohammad Ali Sahraian**

Iran

**Youichi Saitoh**

Japan

**Koichi Sakakura**

United States of America

**Leanne Sakzewski**

Australia

**Mohammad Salajegheh**

United States of America

**Johnny Salameh**

United States of America

**Richard Salazar**

United States of America

**Olivier Salvado**

Australia

**Zainab Samaan**

Canada

**Pascual Sanchez-Juan**

Spain

**Howard Sander**

United States of America

**Thomas Sanderson**

United States of America

**Isabel Santana**

Portugal

**Filippo M. Santorelli**

Italy

**Sophie Sarre**

United Kingdom

**Taeko Sasai-Sakuma**

Japan

**Jaume Sastre-Garriga**

Spain

**Giovanni Savettieri**

Italy

**Kittisak Sawanyawisuth**

Thailand

**Christoph Schankin**

Germany

**Martin Schecklmann**

Germany

**Jan Friedrich Scheitz**

Germany

**Ben Schmand**

Netherlands

**Holger Schmidt**

Germany

**J. Michael Schmidt**

United States of America

**Michael Schneck**

United States of America

**Christiane Schneider-Gold**

Germany

**Jean Schoenen**

Belgium

**Christoph Schrader**

Germany

**Jon Schrock**

United States of America

**Markus Schubert**

Germany

**Walter Schulz-Schaeffer**

Germany

**Isabella Schwartz**

Israel

**Giorgio Scivoletto**

Italy

**Rod Scott**

United Kingdom

**Thomas Scott**

United States of America

**Kevin Seburn**

United States of America

**Helen Mary Seeley**

United Kingdom

**Cornelia Seitz**

Germany

**Rudiger Seitz**

Germany

**Arjune Sen**

United Kingdom

**Marco Senzolo**

Italy

**Zbigniew Serafin**

Poland

**Reza Seyedsadjadi**

United States of America

**Eugene Shapiro**

United States of America

**Manu Sharma**

Germany

**Jingpu Shi**

China

**Norihito Shimamura**

Japan

**Won-Seob Shin**

Korea, South

**Yukito Shinohara**

Japan

**Mohamed Shoukri**

Saudi Arabia

**Bob Siegerink**

Netherlands

**Brian Silver**

United States of America

**Robert Simpson**

United Kingdom

**Andrew Slivka**

United States of America

**Derek Sloan**

Malawi

**David Smith**

United Kingdom

**Todd Smitherman**

United States of America

**A Lynn Snow**

United States of America

**Tomás Sobrino**

Spain

**Claudia Sommer**

Germany

**Panupan Songcharoen**

Thailand

**Virawudh Soontornniyomkij**

United States of America

**Carolina Soriano-Tárraga**

Spain

**Gabriela Spulber**

Sweden

**Jonatan Myrup Staalsø**

Denmark

**Maria Stamelou**

Greece

**Nada Starcevic Cizmarevic**

Croatia

**Hermann Stefan**

Germany

**Sherman Stein**

United States of America

**Egon Stenager**

Denmark

**Erwin Stolz**

Germany

**Herwig Strik**

Germany

**Adam Strzelczyk**

Germany

**Sheng-Feng Sung**

Taiwan

**Rainer Surges**

Germany

**Yasuhiro Suzuki**

Japan

**Elisabeth Svensson**

Sweden

**Pille Taba**

Estonia

**Hisao Tachibana**

Japan

**Naoko Tachibana**

Japan

**Björn Tackenberg**

Germany

**Fernanda Tagliaferri**

Italy

**Koji Takano**

Japan

**Ek Tan**

Slovenia

**Wai Kwong Tang**

Hong Kong

**Christian Tanislav**

Germany

**Robert Taylor**

United States of America

**Gioacchino Tedeschi**

Italy

**Jose Francisco Tellez-Zenteno**

Canada

**Mario Teo**

United States of America

**Niccolo Terrando**

Sweden

**Alessandro Tessitore**

Italy

**Mark Thaller**

United Kingdom

**Aurore Thibaut**

Belgium

**Vincent Thijs**

Belgium

**Katja Thomas**

Germany

**Vincent Tiffreau**

France

**Vincent Timmerman**

Belgium

**Amir. M Torabi**

United States of America

**Helen Tremlett**

Canada

**Maria Trojano**

Italy

**Nina Tsakadze**

United States of America

**Benjamin Tseng**

United States of America

**Georgios Tsivgoulis**

Greece

**Antonino Tuttolomondo**

Italy

**Shun-Fen Tzeng**

Taiwan

**Akira Uchino**

Japan

**Tomas Uher**

Czech Republic

**Christina Ulane**

United States of America

**Anetta Undas**

Poland

**Randall Urban**

United States of America

**Kimiaki Utsugisawa**

Japan

**Thiago Vale**

Brazil

**Annamaria Vallelunga**

Italy

**Nens Van Alfen**

Netherlands

**Janneke Van Beijnum**

Netherlands

**Ingrid Van De Port**

Netherlands

**Peter Van Den Bergh**

Belgium

**Arn Van Den Maagdenberg**

Netherlands

**Quinten Van Geest**

Netherlands

**Neeltje Van Haren**

Netherlands

**Caroline Van Heugten**

Netherlands

**Joost Van Middendorp**

United Kingdom

**I.N. Van Schaik**

Netherlands

**Gregory Van Stavern**

United States of America

**Paulette Van Vliet**

Australia

**Nora Vanegas**

United States of America

**Euthymia Vargiami**

Greece

**Carlos Vasconcelos**

Portugal

**Brandon Vasquez**

Canada

**Cecilia Vecoli**

Italy

**László Vécsei**

Hungary

**Pierangelo Veggiotti**

Italy

**Latha Velayudhan**

United Kingdom

**André Venter**

South Africa

**Joe Verghese**

United States of America

**Meike Vernooij**

Netherlands

**Carles Vilarino-Guell**

Canada

**Simona Villani**

Italy

**Anand Viswanathan**

United States of America

**Eleonora Volpato**

Italy

**Michael Von Brevern**

Germany

**F. Rainer Von Coelln**

United States of America

**Lena Von Koch**

Sweden

**Richard Walker**

United Kingdom

**Allan Walkey**

United States of America

**Mark Walterfang**

Australia

**Daowen Wang**

China

**Jin-Hui Wang**

China

**Pn Wang**

United Kingdom

**Lijuan Wang**

China

**Mohammad Wasay**

Pakistan

**Carina Wattmo**

Sweden

**Alastair Webb**

United Kingdom

**Martin Weber**

Germany

**Nico Weerkamp**

Netherlands

**Bianca Weinstock-Guttman**

United States of America

**Paul Welsh**

United Kingdom

**David Werring**

United Kingdom

**Wendy Westbroek**

United States of America

**Willeke Westendorp**

Netherlands

**Kirk Wilhelmus**

United States of America

**Joshua Willey**

United States of America

**Anders Wimo**

Sweden

**Gavin Winston**

United Kingdom

**Christian Wöber**

Austria

**Edwina Wright**

Australia

**Ruey-Meei Wu**

Taiwan

**Ming Wu**

United States of America

**Yu-Chung Wu**

Taiwan

**Yih-Ru Wu**

Taiwan

**Zhang Xi**

China

**Jianfeng Xiao**

United States of America

**Bing Xing**

China

**Yunyun Xiong**

China

**Yad Ram Yadav**

India

**Yoshihisa Yamano**

Jordan

**Yue Yang**

United States of America

**Gordon Sk Yau**

Hong Kong

**Leonard Yeo**

Singapore

**So-Mei (Teresa) Yeung**

Canada

**Jonas Yeung**

Hong Kong

**Michael Yoong**

United Kingdom

**Asako Yoritaka**

Japan

**Tatsuki Yoshimatsu**

Japan

**Hideyuki Yoshioka**

Japan

**Jin-Tai Yu**

China

**Rey-Yuan Yuan**

Taiwan

**Asma Zakaria**

United States of America

**Konstantine Zakzanis**

Canada

**Mauro Zampolini**

Italy

**Michael Zandi**

United Kingdom

**Ali Zandieh**

Iran

**Peifeng Zhang**

China

**Xinfu Zhou**

Austria

**Jasna Zidverc-Trajkovic**

Serbia

**Zvinka Zlatar**

United States of America

